# Antiproliferative activity, cell-cycle arrest, apoptotic induction and LC-HRMS/MS analyses of extracts from two *Linum* species

**DOI:** 10.1080/13880209.2022.2102196

**Published:** 2022-08-09

**Authors:** Ryma Mouna, Alexis Broisat, Abdalwahab Ahmed, Marlène Debiossat, Ahcène Boumendjel, Catherine Ghezzi, Zahia Kabouche

**Affiliations:** a Université des frères Mentouri-Constantine 1, Laboratoire d'Obtention de Substances Thérapeutiques (LOST), Constantine, Algeria; b Université de Grenoble Alpes, INSERM, CHU Grenoble Alpes, LRB, Grenoble, France; c Department of Chemistry, College of Science, Sudan University of Science and Technology, Khartoum, Sudan

**Keywords:** *L. numidicum* Murb., *Linum trigynum* L., anticancer activity, phytochemical profiling

## Abstract

**Context:**

*Linum* is the largest genus of the Linaceae family; the species of this genus are known to have anticancer activity.

**Objective:**

In this study, ethyl acetate extracts of *L. numidicum* Murb. (EAELN) and *L. trigynum* L. (EAELT) were examined, for the first time, for their anticancer capacity. The secondary metabolites compositions were analysed by LC-HRMS/MS.

**Materials and methods:**

The antiproliferative effect of EAELN and EAELT (0–10.000 μg/mL) against PC3 and MDA-MB-231 cell lines were  evaluated by the MTT assay after 72 h of treatment. Flow cytometer analysis of apoptosis (Annexin V-FITC/PI) and cell cycle (PI/RNase) was also performed after treatment with EAELN and EAELT at 250, 500, and 1000 μg/mL, for 24 h.

**Results:**

EAELN had the highest antiproliferative activity against PC3 (IC_50_ 133.2 ± 5.73 μg/mL) and MDA-MB-231 (IC_50_ 156.9 ± 2.83 μg/mL) lines, EAELN had also shown better apoptotic activity with 19 ± 2.47% (250 μg/mL), 87.5 ± 0.21% (500 μg/mL), and 92 ± 0.07% (1000 μg/mL), respectively, causing cell cycle arrest of PC3 cells in G2/M phase, whereas arrest in G0/G1 and G2/M phases was observed after treatment with EAELT. LC-HRMS/MS profiling of the extracts revealed the presence of known compounds that might be responsible for the observed anticancer activity such as chicoric acid, vicenin-2, vitexin and podophyllotoxin-β-d-glucoside.

**Discussion and conclusions:**

We have shown, for the first time, that EAELN and EAELT exert anticancer activity through cell cycle arrest and induction of apoptosis. EAELN can be considered as a source to treat cancer. Further studies will be required to evaluate the effect of the active compounds, once identified, on other cancer cell lines.

## Introduction

Cancer is the second main cause of death in the world and remains one of the most difficult diseases to combat (Teles et al. 2018). Cancer is characterised by the uncontrolled proliferation of cells, loss of cell cycle control and insensitivity to apoptosis which often lead to the formation of malignant tumours, which can invade neighbouring parts of the organism (Seyfried and Shelton 2010).

The development of anticancer drugs and more effective treatment strategies to improve the quality of life of patients is of great importance in the field of oncology. Currently, cancer treatments, such as chemotherapy and radiotherapy have drawbacks, including strong systemic toxicities and local irritations (Shibata et al. 1990; Huncharek et al. 2001). In addition, resistance to anticancer drugs and adverse outcomes of radiotherapy (Jabir et al. 2018; Lee et al. 2018) emphasise the urgent need to discover new, less toxic agents with higher clinical efficacy (Lee et al. 2003; Newman and Cragg 2007).

Medicinal plants are known for constituting a rich source of clinically relevant anticancer compounds. In this context, the inhibition of cancer cell proliferation and induction of apoptosis by phytochemicals is considered a promising feature of chemotherapeutic drugs (Lowe and Lin 2000; Gurumurthy et al. 2001). Currently, many major anticancer drugs available are either natural products or their derivatives, such as taxol, vinblastine, vincristine, and camptothecin (Newman et al. 2003; Newman and Cragg 2007; Ojima 2008).

The *Linum* genus (Linaceae) includes more than 200 species distributed throughout the world (Rogers 1982). Several studies have reported that *Linum* species might inhibit the growth of various types of cancer cell lines through cell cycle arrest and induction of apoptosis (Amirghofran et al. 2006; Mohammed et al. 2010; Alejandre-García et al. 2015; Akbari Asl et al. 2018). The investigation of the phytochemical composition of *Linum* species revealed the presence of lignan-type compounds (Vasilev et al. 2008). These types of compounds constitute an important group of natural products that exert different biological activities and can serve as lead compounds for the development of new therapeutic agents with antiangiogenic, antirheumatic, antipsoriasis, antiasthmatic, hypolipidemic, antifungal, and antiviral activity (Ayres and Loike 1990; Iwasaki et al. 1996). Moreover, cytotoxic and antitumor activities are of major interest for these types of lignans (Ayres and Loike 1990).

The present study determines the ability of the ethyl acetate extracts (EAELN and EAELT) of two Algerian *Linum* species, *L. numidicum* Murb. and *L. trigynum* L., respectively, to inhibit cancer cell proliferation, block the cell cycle and induce apoptosis. In addition, the secondary metabolites composition of the two extracts was analysed by LC-HRMS/MS to determine the relationship between their anticancer activity and their chemical composition.

According to the World Health Organisation (WHO) 2020, the highest incidences in the world are observed for breast and prostate cancers. We, therefore, decided, as proof of concept, to test the effect of our extracts on breast and prostate cancer cell lines.

## Materials and methods

### Preparation of ethyl acetate extracts of *L. numidicum* and *L. trigynum*

*L. numidicum was* collected in June 2018 from Djebel Babor in Sétif (North-Eastern Algeria), while the collection of *L. trigynum* was made in June 2017 from the region of Djebel El-Ouahch in Constantine (North-Eastern Algeria). The two plants used in the study were authenticated by Prof. Gérard de Bélair (ENSA, El-Harrach, Algeria). They are registered in the herbarium of the Laboratory LOST at University Mentouri Constantine 1 with the respective Voucher codes LOST.LN.06.18 and LOST.LT.06.17.

They are also registered at the national herbarium GdB: https://gdebelair.com/tax/zzeo.html#Linaceae

Id: 077_44, Taxon: Linaceae*, Linum numidicus* Murbeck, Coordinates: *x* = 5.455072, *y* = 36.493397, *z* = 1855 m, Locality: Djebel Babor (Sétif)
Id: 011_42, Taxon: Linaceae*, Linum trigynum* L., Coordinates: *x* = 6.66415, *y* = 36.403014, *z* = 970 m, Locality: Djebel El Ouahch (Constantine)

The aerial parts of *L. numidicum* and *L. trigynum* (900 g), previously dried and pulverised, were individually macerated at room temperature in a hydroalcoholic mixture (EtOH/H_2_O, 8:2, v/v) for a period of 72 h. The crude extracts obtained were taken up with distilled water (1000 mL). The latter underwent liquid/liquid type extractions, using solvents of increasing polarity (petroleum ether, chloroform, ethyl acetate and *n*-butanol). The obtained organic phases were concentrated under reduced pressure to dryness.

### Sample preparation

Dried acetate extracts from both plants (EAELN and EAELT) were dissolved in dimethylsulphoxide (DMSO, Sigma Aldrich) followed by an RPMI medium. The DMSO content in the solution did not exceed 0.1%. The mixture was then centrifuged to remove insoluble ingredients and the supernatant was passed through sterilisation filters. The solution was diluted with an RPMI medium and prepared at different concentrations.

### Cell culture

MDA-MB-231 cells, derived from pleural effusion of breast adenocarcinoma, from ATCC, were cultured in DMEM (PAN Biotech; Aidenbach, Germany) supplemented with 10% foetal bovine serum (FBS; Dominique Dutscher, SA; Brumath, France) and 1% penicillin/streptomycin. The PC3 prostate cancer line from ATCC was cultured using RPMI 1640 medium (PAN Biotech; Aidenbach, Germany) supplemented with 10% FBS and 1% penicillin/streptomycin. The cell lines were grown in an incubator at 37 °C with 5% CO_2_ and 95% humidity.

### MTT test

The antiproliferative activity of EAELN and EAELT against the two selected cell lines was evaluated by the MTT assay (3-[4,5-dimethylthiazol-2-yl]-2,5-diphenyltetrazolium bromide; Sigma-Aldrich), according to Mossman (1983). PC3 and MDA-MB-231 lines (5000 cells/well) were seeded in 96-well plates and incubated with different concentrations of EAELN and EAELT (2.44, 9.77, 39.06, 156.25, 625, 2500, and 10.000 μg/mL). Cells without treatment were used as controls. After 72 h of exposure to the extracts, the wells were emptied, washed with PBS and then 100 µL of MTT (5 mg/mL) was added to each well. The plates were then incubated for an additional 2 h at 37 °C and 5% CO_2_. After that, the medium was carefully aspirated and the formed formazan crystals were dissolved in DMSO. The absorbance in each well was measured at 570 nm using a microplate reader (Varioskan LUX, Thermo Scientific).

### Apoptosis assay

Cell apoptosis induced by EAELN and EAELT was detected by Annexin V-FITC/PI staining according to the manufacturer's instructions (BD Pharmingen TM FITC Annexin V Apoptosis Detection kit I). PC3 cells (10^5^ cells/well) were treated with EAELN and EAELT after seeding in 96-well plates with different concentrations (250, 500, and 1000 µg/mL) for 24 h. Cells were then washed with PBS buffer, centrifuged, and stained with 5 μL of Annexin V and 5 μL of propidium iodide (PI). After 15 min of incubation at room temperature, 400 µL of binding buffer was added. Stained cells were determined using Accuri TM-C6 flow cytometry (BD-Biosciences).

### Cell cycle analysis

The cell cycle was determined using a cell cycle detection kit (BD Pharmingen TM PI/RNase Staining Buffer). PC3 cells (10^6^ cells/well) were treated with EAELN and EAELT after seeding in 6-well plates with different concentrations (250, 500, and 1000 µg/mL) for 24 h. At the end of the treatment, cells were collected and washed with cold PBS and fixed in cold 70% ethanol (−20 °C) for 2 h. The cells were then centrifuged and the cell pellet was resuspended with 0.5 mL of a mixture containing propidium iodide and RNase. After 15 min of incubation at room temperature, the DNA content of the cells was quantified using an Accuri TM-C6 flow cytometer (BD-Biosciences).

### LC-HRMS/MS analyses

LC − ESI − DDA − HRMS/MS spectra were obtained using a Dionex Ultimate 3000 liquid chromatography (HPLC) system equipped with an Ultimate 3000 RS pump hyphenated to a Thermo Instruments MS system (LTQ Orbitrap XL). The HPLC analysis was performed on a Water BEH C18 column (1.7 μm, 2.1 mm × 150 mm) and using the following solvents: Solvent A 0.1% formic acid H_2_O, Solvent B 0.1% formic acid acetonitrile. The gradient used was 5% B for 5 min, 5–100% for 20 min, and 100% for 7 min. The flow rate used was 0.150 mL/min and the column temperature was at 45 °C. MS/MS spectra were realised using MZmine 2.53 with a mass detection noise level set at 2E4 and 2E, respectively in both positive and negative ion modes. The compound identification was aided by using the Global Natural Product Social Molecular Networking platform (GNPS).

### Statistical analyses

Data analyses were performed using GraphPad PRISM software (version 8.0.2). All experiments were performed in triplicate. One-way or 2-way ANOVA with Bonferroni’s *post hoc* comparisons was used as indicated in the legends. Data are presented as mean ± SD. Significant differences were set at a *p*-value < 0.05.

## Results

### Antiproliferative effect of EAELN and EAELT on the PC3 and MDA-MB-231 cell lines

To study the effect of EAELN and EAELT on the growth of human prostate and breast cancer cells, PC3 and MDA-MB231 cells were treated with different concentrations of extracts for 72 h, and cell viability was determined using the MTT assay.

**Figure 1.**
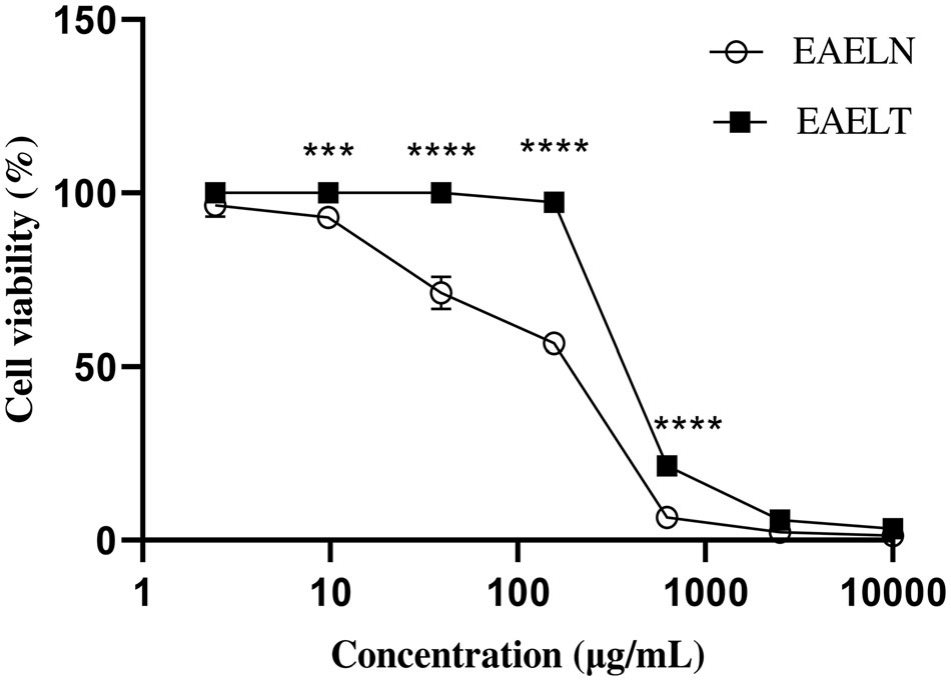
Dose-dependent effect of EAELN and EAELT on the viability of PC3 cancer cells. Cell viability was determined by an MTT assay and was expressed as a percentage. Cells were treated with tow extracts at different concentrations for 72 h. Data are expressed as mean ± SD (*n* = 3), ***p < 0.001, ****p < 0.0001, EAELN versus EAELT. Two-way ANOVA followed by Bonferroni’s correction.

**Figure 2.**
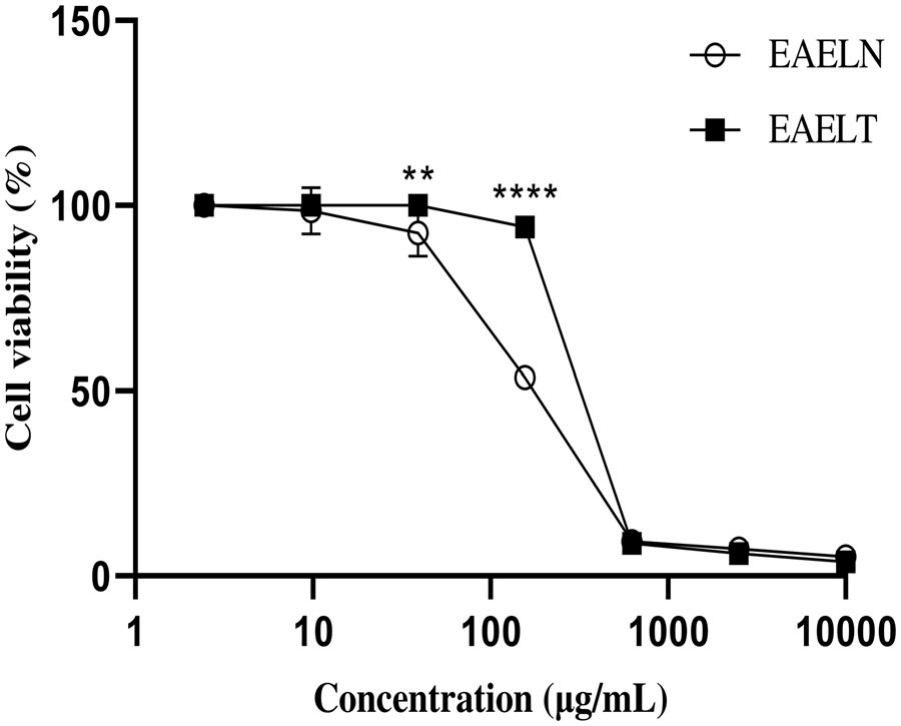
Dose-dependent effects of EAELN and EAELT on the viability of MDA-MB-231 cancer cells. Cell viability was determined by an MTT assay and was expressed as a percentage. Cells were treated with tow extracts at different concentrations for 72 h. Data are expressed as mean ± SD (*n* = 3), **p < 0.01, ****p < 0.0001, EAELN versus EAELT, Two-way ANOVA followed by Bonferroni’s correction.

MTT assay results indicated that EAELN and EAELT inhibited the proliferation of PC3 and MDA-MB-231 cells in a concentration-dependent manner (Figures 1 and 2), with semi-maximal inhibitory concentration (IC_50_) values of 133.2 ± 5.73 μg/mL and 156.9 ± 2.83 μg/mL, respectively, for EAELN and 415.8 ± 2.33 μg/mL and 307.5 ± 2.97 μg/mL, respectively, for EAELT (Table 1).

**Table 1.** IC_50_ values (μg/mL) of ethyl acetate extracts of *L. numidicum* (EAELN) and *L. trigynum* (EAELT) against the prostate cancer line (PC3) and the breast adenocarcinoma line (MDA-MB-231).

Cell type	IC_50_ values (μg/mL)	
	EAELN	EAELT
PC3	133.2 ± 5.73	415.8 ± 2.33
MDA-MB-231	156.9 ± 2.83	307.5 ± 2.97

Data are presented as mean ± SD of IC_50_ (µg/mL) from three independent experiments.

EAELN, Ethyl acetate extract of *L. numidicum*; EAELT, Ethyl acetate extract of *L. trigynum*.

### Induction of apoptosis by EAELN and EAELT in PC3 cells

To explore the mechanism by which EAELN and EAELT inhibit cell proliferation, a flow cytometer apoptosis analysis, using Annexin V-FITC/PI double staining, was performed after a 24 h exposure period to different extract concentrations (250, 500, and 1000 μg/mL).

**Figure 3.**
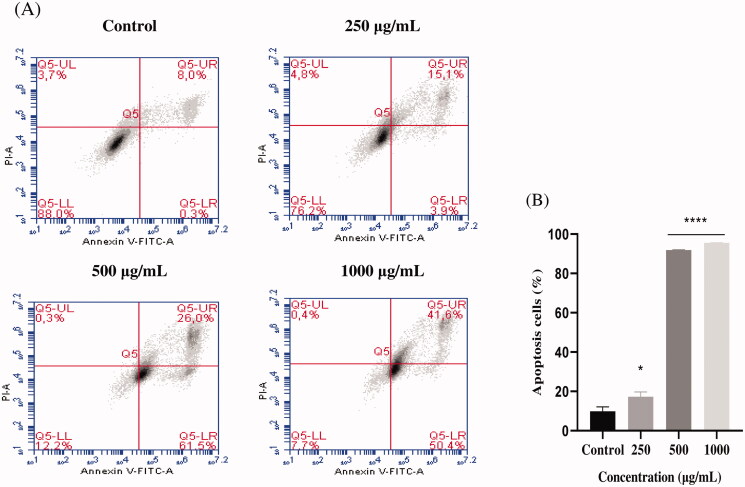
Induction of apoptosis by EAELN in PC3 cancer cells. (A) Representative histograms of cells sorted by flow cytometry. Cells were treated with the extract for 24 h at different concentrations (250, 500, and, 1000 μg/mL) and stained with Annexin V-FITC and propidium iodide before sorting by flow cytometry. Cells in the Q5-UL, Q5-UR, Q5-LL and Q5-LR quadrants represent necrotic, late apoptotic, viable and early apoptotic populations, respectively. (B) Quantification of apoptotic cells. The percentage of apoptotic cells was calculated. Each bar represents the mean ± SD (*n* = 3), *p < 0.05, ****p < 0.0001, compared with control. One-way ANOVA followed by Bonferroni’s correction.

**Figure 4.**
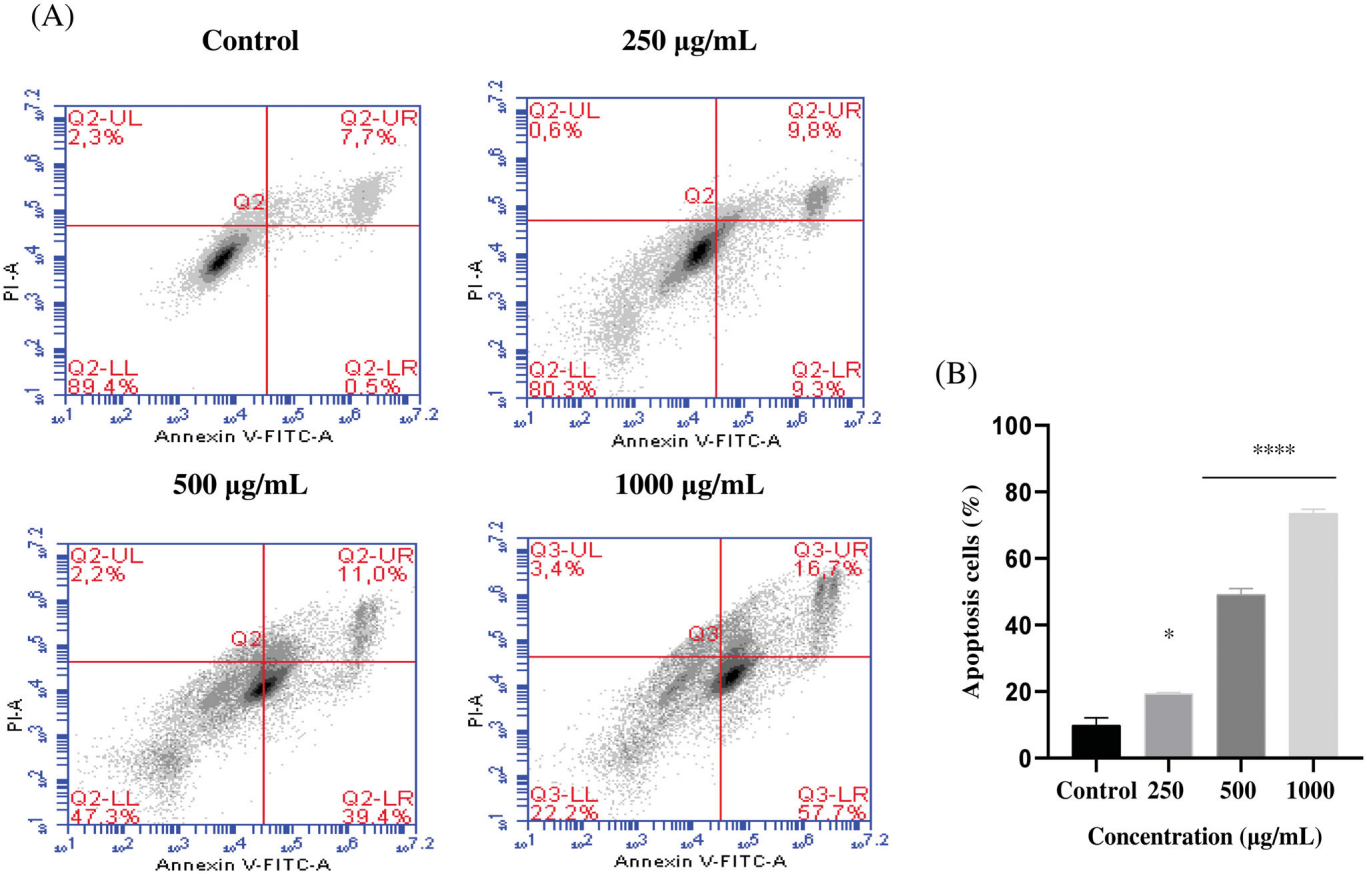
Induction of apoptosis by EAELT in the PC3 cancer cell. (A) Representative histograms of cells sorted by flow cytometry. Cells were treated with the extract for 24 h at different concentrations (250, 500, and, 1000 μg/mL) and stained with Annexin V-FITC and propidium iodide before sorting by flow cytometry. Cells in the Q5-UL, Q5-UR, Q5-LL and Q5-LR quadrants represent necrotic, late apoptotic, viable and early apoptotic populations, respectively. (B) Quantification of apoptotic cells. The percentage of apoptotic cells was calculated. Each bar represents the mean ± SD (*n* = 3), *p < 0.05, ****p < 0.0001, compared with control. One-way ANOVA followed by Bonferroni’s correction.

EAELN and EAELT induced apoptosis of PC3 cells, which was evidenced by the accumulation of early and late apoptotic cells, and by the decrease of viable cells, with a higher effect at a dose of 500 and 1000 μg/mL (****p < 0.0001) compared with the negative control (Figures 3A,B and 4A,B).

The proportion of total apoptotic cells (early and late) after treatment with EAELN increased from 8.3 ± 2.26% (control) to 19 ± 2.47% (250 μg/mL), 87.5 ± 0.21% (500 μg/mL), and 92 ± 0. 07% (1000 μg/mL), respectively, and after treatment with EAELT, the proportion of total apoptotic cells increased from 8.2 ± 2.33% (control) to 19.1 ± 0.35% (250 μg/mL), 50.4 ± 1.77% (500 μg/mL), and 74.4 ± 1.27% (1000 μg/mL), respectively.

### Induction of cell cycle arrest in PC3 cells by EAELN and EAELT

To better understand the mechanism by which EAELN and EAELT inhibit cell proliferation, flow cytometry analysis using PI staining was performed to assess cell cycle distribution in the PC3 cell line after 24 h of exposure to 250, 500, and 1000 μg/mL of extract. After treatment with EAELN, the percentage of cells in the G0/G1 and S phase was reduced, while that in the G2/M phase was significantly increased (****p < 0.0001).

**Figure 5.**
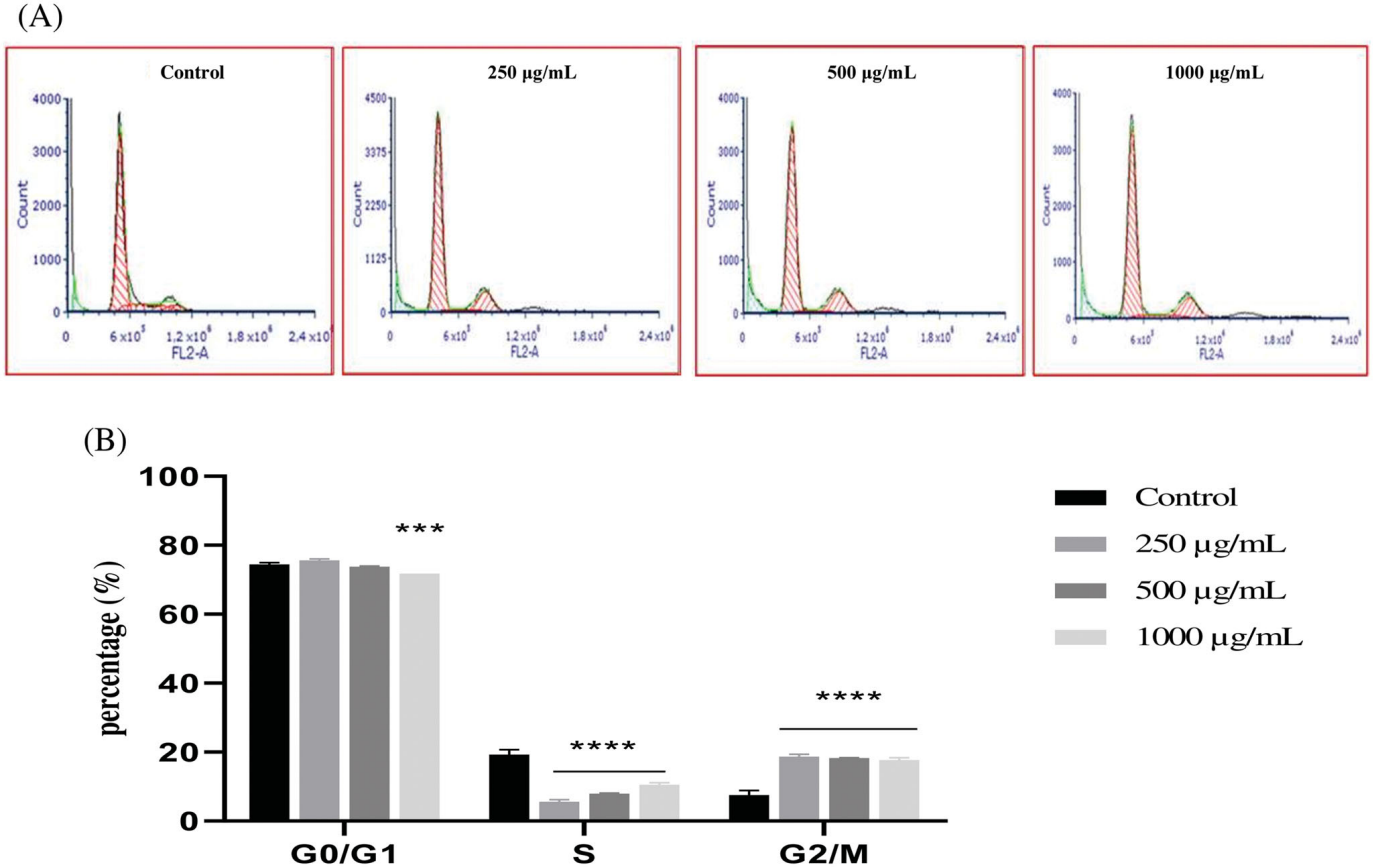
The effect of EAELN on cell cycle distribution in PC3 cell lines. The PC3 cell line was incubated for 24 h with the extract at different concentrations (250, 500 and 1000 μg/mL). (A) Flow cytometry analysis of cell distribution is represented by PI fluorescence histograms. The experiment was repeated three times. (B) The percentages of cells in the different phases are shown in the bar graph. With increasing extract concentration, the number of cells in the G2/M phase increased while that in the G0/G1 and S phase decreased. EAELN blocks cells in the G2/M phase. Data are presented as means ± SD (*n* = 3), ***p < 0.001, ****p < 0.0001, compared with control. Two-way ANOVA followed by Bonferroni’s correction.

**Figure 6.**
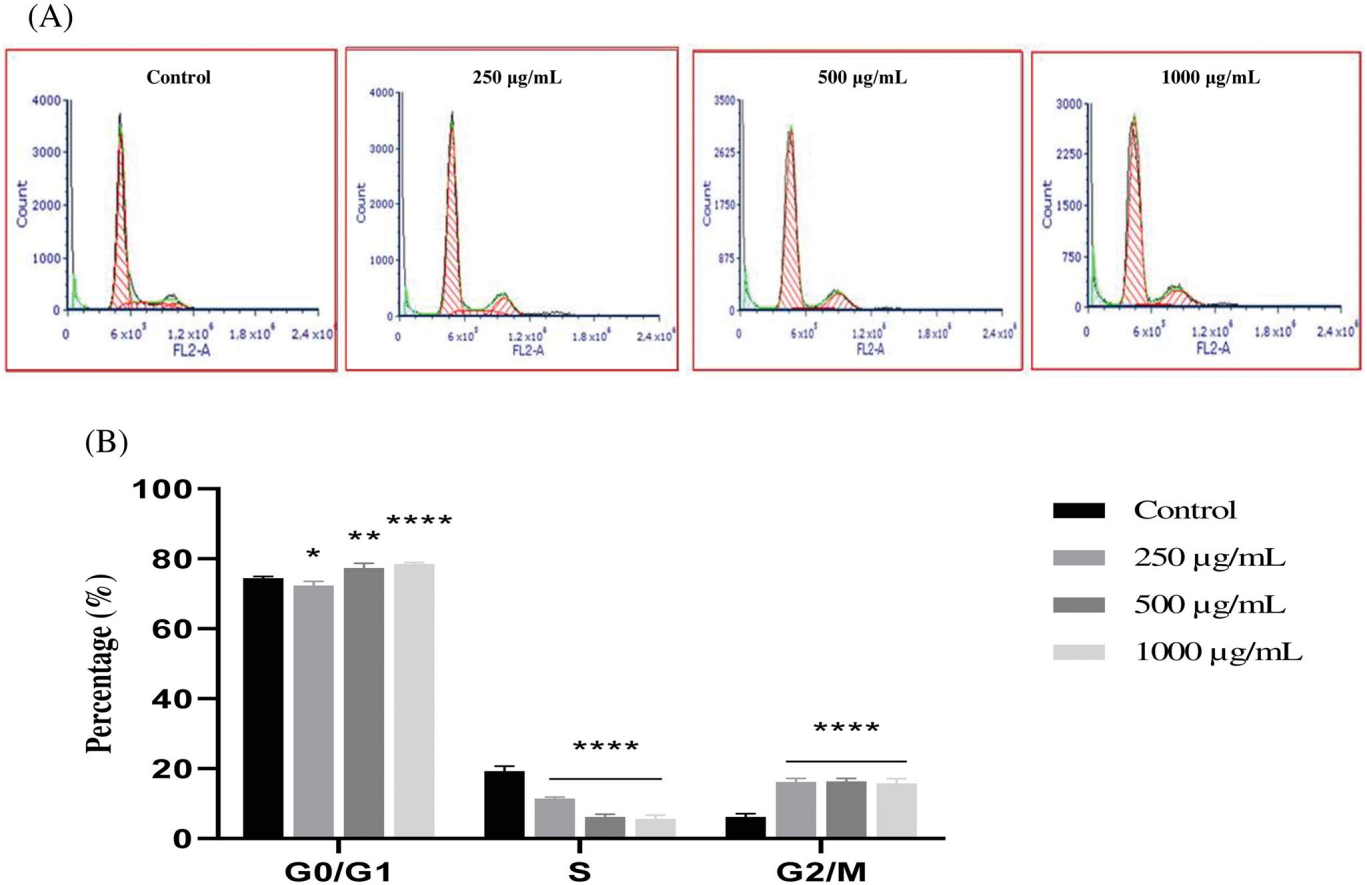
The effect of EAELT on cell cycle distribution in PC3 cell lines. The PC3 cell line was incubated for 24 h with the extract at different concentrations (250, 500, and 1000 μg/mL). (A) Flow cytometry analysis of cell distribution is represented by PI fluorescence histograms. The experiment was repeated three times. (B) The percentages of cells in the different phases are shown in the bar graph. With increasing extract concentration, the number of cells in the G0/G1 and G2/M phase increased while that in the S phase decreased. EAELT extracts blocked cells in G0/G1 and G2/M phases. Data are presented as means ± SD (*n* = 3), *p < 0.05, **p < 0.01, ****p < 0.0001, compared with control. Two-way ANOVA followed by Bonferroni's correction.

The percentage of cells in the G0/G1 phase in the three groups of samples treated with EAELN was 75.60 ± 0.47%, 73.75 ± 0.22%, and 71.79 ± 0.01 (***p < 0.001) for 250, 500, and 1000 μg/mL, respectively, while that of the control was 74.46 ± 0.49%. The percentage of cells in S phase increased from 19.29 ± 1.39% (control) to 5.66 ± 0.53% (250 μg/mL), 7.96 ± 0.20% (500 μg/mL), and 10.55 ± 0.55 (1000 μg/mL), all ****p < 0.0001 compared with control, while that in G2/M phase went from 7.50 ± 1.36% (control) to 18.73 ± 0.67% (250 μg/mL), 18.29 ± 0.12% (500 μg/mL), and 17.72 ± 0.64% (1000 μg/mL), all *p* < 0.0001 compared with control (Figure 5A, B). Treatment with EAELT showed different effects in different phases of the cell cycle, with the percentage of cells in the S phase being reduced, while that of G0/G1 and G2/M phase increased significantly (Figure 6A, B).

The percentage of cells in G0/G1 phase after EAELT treatment increased from 74.46 ± 0.49% (control) to 72.35 ± 1.20% (250 μg/mL, *p < 0.05), 77.36 ± 1.38% (500 μg/mL, **p < 0.01) and 78.58 ± 0.37% (1000 μg/mL, ****p < 0.0001), respectively, and that of the G2/M phase increased from 6.25 ± 0.92% (control) to 16.23 ± 0.97% (250 μg/mL), 16.39 ± 0.79% (500 μg/mL), and 15.80 ± 1.32% (1000 μg/mL), respectively, all ****p < 0.0001 compared with control, whereas the percentage of cells in S phase was significantly reduced (****p < 0.0001) from 19. 29 ± 1.39 (control) to 11.42 ± 0.44 (250 μg/mL), 6.25 ± 0.68 (500 μg/mL), and 5.62 ± 1.1 (1000 μg/mL) (Figure 6A, B). The results showed that EAELN induced a significant cell cycle arrest in the G2/M phase, while an arrest in the G0/G1 and G2/M phases was observed in PC3 cells after exposure to various concentrations of EAELT, for 24 h.

### LC-HRMS/MS analyses of EAELN and EAELT

**Figure 7.**
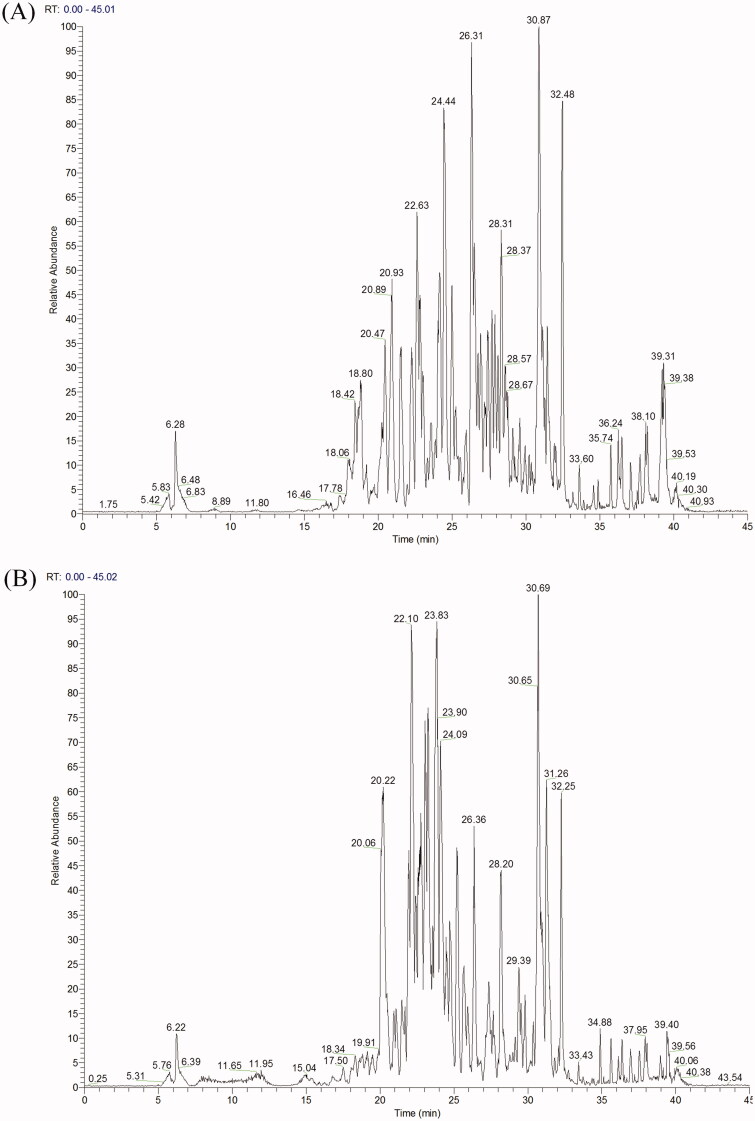
(A) LC − ESI − DDA − HRMS base peak chromatogram of EAELN in negative ion mode. (B) LC − ESI − DDA − HRMS base peak chromatogram of EAELT in negative ion mode.

With the aim to analyse the phytochemical composition of EAELN and EAELT we conducted UHPLC-DAD-ESI/HRMS-MS. The latter revealed the presence of 75 compounds (Table 2 and Figure 7A, B) in each extract. The metabolites were identified using the GNPS platform. EAELN and EAELT showed the presence of polyphenols, terpenoids, alkaloids, polyketides, fatty acids, and carbohydrates. The most important classes were flavones (23 compounds) and hydroxycinnamic acids (17 compounds).

**Table 2.** Compounds characterised by LC-RHMS/MS analyses of ethyl acetate extracts of *L. numidicum* (EAELN) and *L. trigynum* (EAELT).

No.	Compounds	Rt (min)	m/z (molecular ion)	Intensity (EAELN)	Intensity (EAELT)
1	Melibiose	4.99	365.1138	147 929 531 (3.97%)	147 978 181 (2.36%)
2	Chlorogenic acid	18.02	355.1008	19 830 953 (0.53%)	466 338 325 (7.44%)
3	Caffeoylquinic acid	18.4	353.0862	2 047 497 (0.05%)	76 620 264 (1.22%)
4	Vicenin-2 isomer1	18.94	595.1646	300 955 606 (8.08%)	219 732 570 (3.50%)
5	Feruloyltyramine	19.07	265.1540	157 369 (0.004%)	0
6	3-*O*-Coumaroylquinic acid isomer 1	19.17	339.1057	1 531 302 (0.04%)	77 287 147 (1.23%)
7	Melilotoside	19.2	371.0967	21 984 718 (0.59%)	19 742 885 (0.31%)
8	2ʺ-*O*-β-d- Xylopyranosylorientin	19.70	581.1491	429 950 (0.01%)	253 972 376 (4.05%)
9	Methyl chlorogenate isomer 1	20.30	369.1171	2 903 253 (0.08%)	185 972 948 (2.97%)
10	4-Caffeoylquinic acid	20.4	353.0863	32 305 715 (0.87%)	658 912 290 (10.51%)
11	7,2′-Dihydroxyflavone	20.5	253,0708	19 377 588 (0.52%)	7 639 462 (0.12%)
12	Vicenin-2 isomer 2	20.7	593,1488	176 871 262 (4.75%)	2 946 784 (0.05%)
13	3-*O*-Feruloylquinic acid isomer 1	21.0	367,1017	1 330 959 (0.04%)	174 112 107 (2.78%)
14	Lanicepside B	21.36	561.1933	683 831 (0.02%)	1 511 690 (0.02%)
15	Olivil 4*′*-*O*-β-d-glucoside	21.5	583.2010	2 205 970 (0.06%)	1 187 665 (0.02%)
16	Vicenin-2 isomer 3	21.6	593.1488	173 733 674 (4.67%)	108 801 687 (1.74%)
17	5,3*′*-Dihydroxyflavone	21.7	253.0708	104 431 411 (2.80%)	30 222 840 (0.48%)
18	Coumaroyl-*O*-β-d-fructofuranosyl-α-d-glucopyranoside	21.8	487.1436	0	10 332 606 (0.16%)
19	3-*O*-Coumaroylquinic acid isomer 2	22.05	337.0916	12 392 332 (0.33%)	290 472 234 (4.63%)
20	Luteolin-7,3′-di-*O*-β-d-glucoside	22.1	609.1438	4 642 826 (0.12%)	2 201 017 (0.04%)
21	Vicenin-2 isomer 4	22.4	593.1488	192 586 754 (5.17%)	9 952 840 (0.16%)
22	Methyl chlorogenate isomer 2	22.44	369.1171	1 751 179 (0.05%)	2 924 904 (0.05%)
23	3-*O*-Feruloylquinic acid isomer 2	22.5	367.1020	3 479 096 (0.09%)	241 714 645 (3.85%)
24	Chicoric acid	22.8	473.0705	305 001 876 (8.19%)	7 778 556 (0.12%)
25	Orientin isomer 1	22.85	449.1066	94 546 115 (2.54%)	138 356 303 (2.21%)
26	*cis*-Zeatin-*O*-glucoside	22.86	382.1849	3 651 063 (0.1%)	37 911 888 (0.60%)
27	Homoorientin	23.0	447.0912	207 820 192 (5.58%)	577 624 518 (9.21%)
28	Quercetin-3-methoxy-3′-*O*-glucoside isomer 1	23.05	479.1173	40 490 115 (1.09%)	16 892 786 (0.27%)
29	Orientin isomer 2	23.2	447.0914	15 986 955 (0.43%)	24 045 527 (0.38%)
30	2-Phenylethyl 3-*O*-(4-carboxy-3-hydroxy-3-methylbutanoyl)-β-d-glucopyranoside	23.40	451.1607	92 875 737 (2.49%)	7 558 907 (0.12%)
31	Tricin 5-glucoside isomer 1	23.45	493.1326	125 814 691 (3.38%)	8 039 417 (0.13%)
32	8-C-Disaccharide genistein	23.53	565.1543	80 548 (0.002%)	426 027 (0.007%)
33	4,5-Dicaffeoylquinic acid isomer 1	23.7	515.1211	45 099 159 (1.21%)	191 156 967 (3.05%)
34	Methyl chlorogenate isomer 3	23.8	367.1020	448 454 (0.01%)	8 051 002 (0.13%)
35	3,4-Dimethoxydalbergione	23.81	285.1220	70 272 437 (1.89%)	20 260 632 (0.32%)
36	Isovitexin	24.10	433.1117	31 735 817 (0.85%)	23 075 317 (0.37%)
37	Vitexin	24.2	431.0966	280 284 714 (7.53%)	183 668 390 (2.93%)
38	Isovitexin 2ʺ-*O*-arabinoside	24.24	563.1388	42 646 (0.001%)	173 177 (0.003%)
39	6-(3-Benzyloxy-2- hydroxypropoxy)-glucuronic acid	24.4	371.0970	263 066 (0.007%)	309 401(0.005%)
40	Kaempferol-7-*O*-hexoside	24.36	447.0915	31 103 555 (0.84%)	28 574 403 (0.46%)
41	Swertisin	24.45	445.1131	124 569 (0.003%)	21 234 562 (0.34%)
42	Vitexin-2ʺ-rhamnoside	24.5	577.1544	1 303 151 (0.03%)	797 971 (0.01%)
43	Hyperoside	24.54	463.0864	23 074 096 (0.62%)	242 440 610 (3.87%)
44	4,5-Dicaffeoylquinic acid isomer 2	24.8	515.1175	41 362 870 (1.11%)	222 450 268 (3.55%)
45	Ellagic acid	24.9	300.9979	309 652 (0.008%)	3 654 427 (0.06%)
46	Violanthin	25.0	577.1542	696 722 (0.02%)	740 181 (0.01%)
47	Rutin	25.0	609.1437	0	365 553 (0.006%)
48	Guajavarin	25.1	433.0761	2 713 340 (0.07%)	27 315 611 (0.44%)
49	Spiraeoside	25.2	463.0858	814 062 (0.02%)	3 077 180 (0.05%)
50	Dihydroinfectopyrone	25.20	267.1197	79 404 224 (2.13%)	15 136 125 (0.24%)
51	Luteolin	25.37	287.0544	97 253 530 (2.61%)	153 192 910 (2.44%)
52	Rosmarinic acid	25.4	359.0753	46 168 506 (1.24%)	31 520 285 (0.50%)
53	Malvidin 3-*O*-β-galactoside	25.44	493.1326	545 776 (0.01%)	1 106 293 (0.02%)
54	Quercetin-3-methoxy-3′-*O*-glucoside isomer2	25.5	477.1020	31 418 799 (0.84%)	24 295 807 (0.39%)
55	5-*O*-β-d-Glucopyranosyl-7-methoxy-3′,4′-dihydroxy-4-phenylcoumarin	25.51	463.1225	1 076 001 (0.03%)	4 154 266 (0.07%)
56	Afzelin	25.52	433.1119	84 417 798 (2.27%)	4 951 453 (0.08%)
57	Luteolin-7-*O*-β-d-glucoside	25.74	449.1066	1 673 218 (0.04%)	9 548 581 (0.15%)
58	Quercitrin	25.9	447.0913	88 111 709 (2.37%)	210 096 500 (3.35%)
59	Isorhamnetin 3-*O*-β-d-galactoside	26.0	477.1019	8 038 853 (0.22%)	20 374 345 (0.32%)
60	8,3′,4′-Trihydroxyflavone-7-*O*-6(6ʺ-*O*-p-coumaroyl﴿-β-d-glucopyranoside	26.3	593.1487	75 785 (0.002%)	0
61	3-*O*-Coumaroylquinic acid isomer 3	26.32	337.0917	551 064 (0.01%)	69 834 551 (1.11%)
62	Foliasalacioside B1	26.50	527.2462	2 494 169 (0.07%)	2 093 539 (0.03%)
63	Genkwanin	26.59	285.0752	10 742 814 (0.29%)	476 146 (0.008%)
64	Cirsiliol	26.60	331.0804	130 903 808 (3.52%)	516 457 072 (8.24%)
65	9-(2,3-Dihydroxypropoxy)-9-oxononanoic acid	26.7	261.1334	4 813 192 (0.13%)	2 570 671 (0.04%)
66	Tricin 5-glucoside isomer 2	26.9	491.1174	16 112 666 (0.43%)	43 903 953 (0.70%)
67	Methoxy-quercetin-3-*O*-hexoside	27.4	477.1016	49 158 (0.001%)	0
68	Blumenol C glucoside	27.44	373.2212	5 616 862 (0.15%)	4 813 282 (0.08%)
69	4-(3,4-Dihydroxy-phenyl)-6,7-dihydroxy-naphthalene-2-carboxylic acid	27.7	311.0551	18 083 364 (0.49%)	4 622 489 (0.07%)
70	Phlorhizin	28.0	435.1288	906 364 (0.02%)	36 104 719 (0.58%)
71	Podophyllotoxin-β-d-glucoside	28.1	621.1804	164 285 959 (4.41%)	299 083 (0.005%)
72	Tetrahydroxyflavone	28.4	285.0394	249 356 019 (6.70%)	273 110 205 (4.36%)
73	6,4′-dimethoxy scutellarein-7-neohesperidiside	29.2	667.1756	0	859 816 (0.01%)
74	Trihydroxyflavone	29.6	269.0404	25 972 811 (0.70%)	4 631 461 (0.07%)
75	3-(2,6-Dihydroxyphenyl)-4-hydroxy-6-methyl benzofuranone	29.8	271.0604	2 623 339 (0.07%)	14 180 305 (0.23%)
76	Lactarorufin A	30.8	265.1453	2 422 100 (0.07%)	26 032 990 (0.42%)
77	12,13-Dihome	33.9	313.2372	1 598 402 (0.04%)	125 993 (0.002%)
78	α-Dimorphecolic acid	34.4	295.2266	9 540 392 (0.26%)	9 397 797 (0.15%)

EAELN, Ethyl acetate extract of *L. numidicum*; EAELT, Ethyl acetate extract of *L. trigynum*; RT, Retention time.

#### Compounds detected in EAELN

Forty flavonoids (flavones, flavonols, isoflavones, neoflavoid, chalcones, and one anthocyanidin) were detected. The major compounds were vicenin-2 isomer 1 (**4**), vitexin (**37**), trihydroxyflavone (**74**), homoorientin (**27**), vicenin-2 isomer 4 (**21**), vicenin-2 isomer 2 (**12**), vicenin-2 isomer 3 (**16**), cirsiliol (**64**), tricin 5-glucoside isomer 2 (**66**), 5,3′-dihydroxyflavone (**17**), luteolin (**51**), orientin isomer 1 (**25**), with respective percentages 8.08, 7.53, 6.70, 5.58, 5.17, 4.75, 4.67, 3.52, 3.38, 2.80, 2.61, and 2.54%. Four lignans, lanicepside B (**14**), olivil 4′*-O*-glucoside (**15**), a phenylnaphthalene (**69**), and the major compound podophyllotoxin-β-d-glucoside (**71**) with a percentage of 4.41%, were detected. In addition, 19 phenolic acids, from which 17 hydroxycinnamic acids and two hydroxybenzoic acids were detected with chicoric acid (**24**) as the major compound (8.19%). The analyses also showed the presence of one phenylethanoid (**30**) and one coumarin derivative (**55**), besides other compounds including one carbohydrate, melibiose (**1**), one alkaloid (**26**), two polyketides (**50** and **75**), three terpenoids (**62**, **68** and **76**), and three fatty acids (**65**, **77** and **78**) (Table 2).

#### Compounds detected in EAELT

Forty flavonoids (flavones, flavonols, isoflavones, neoflavoid, chalcone, and anthocyanidin) were detected with homoorientin (**27**), cirsiliol (**64**), tetrahydroxyflavone (**72**), 2ʺ-*O*-β-d-xylopyranosylorientin (**8**), hyperoside (**43**), vicenin-2 isomer1 (**4**), quercitrin (**58**), and vitexin (**37**) as major compounds with respective percentages of 9.21, 8.24, 4.36, 4.05, 3.87, 3.50, 3.35, and 2.93%. The same lignans (**14**, **15**, **69**, and **71**) as those detected in EAELN, were also found in EAELT together with nineteen phenolic acids (17 hydroxycinnamic acids and two hydroxybenzoic acids) namely, 4-caffeoylquinic acid (**10**), chlorogenic acid (**2**), 3-*O*-coumaroylquinic acid isomer 2 (**19**), 3-*O*-feruloylquinic acid isomer 2 (**23**), 4,5-dicaffeoylquinic acid isomer 2 (**44**), 4,5-dicaffeoylquinic acid isomer 1 (**33**), and methyl chlorogenate isomer 1 (**9**) with respective percentages of 10.51, 7.44, 4.63, 3.85, 3.55, 3.05, and 2.97%. The same other compounds (**30**, **55**, **1**, **26**, **50**, **75**, **62**, **68**, **76**, **65**, **77**, and **78**) (Table 2), belonging to different phytochemical classes, which were detected in EAELN, also characterised the EAELT.

## Discussion

Medicinal plants are important sources for the development of effective anticancer agents. Currently, many drugs available on pharmacy shelves are either natural products or their derivatives (Mukherjee et al. 2001; Khan 2014; Ijaz et al. 2018).

Species of *Linum* genus are known to induce anticancer activity (Hartwell 1982). The present study examined, for the first time, the ability of EAELN and EAELT to inhibit cancer cell proliferation, block the cell cycle and induce apoptosis which is known to be the most promising routes to treat cancer (Hanahan and Weinberg 2000; Ghobrial et al. 2005). In addition, the secondary metabolites of EAELN and EAELT were analysed by LC-HRMS/MS to determine the relationship between their anticancer activity and their chemical composition.

The results of our study showed, for the first time, that EAELN and EAELT inhibited the proliferation of PC3 and MDA-MB-231 cells significantly in a concentration-dependent manner (Figures 1 and 2). This could be explained by the presence of polyphenols such as flavonoids, phenolic acids, and lignans in both extracts. Polyphenols could exert anticancer effects through different mechanisms, including modification of cell signalling, inhibition of cell proliferation, induction of cell cycle arrest, and apoptosis (Spatafora and Tringali 2012; Abbas et al. 2017). Therefore, the current study suggests that polyphenols, detected in the extracts, may exhibit anticancer activity by inducing apoptosis and blocking the cell cycle at different stages. EAELN had the highest antiproliferative activity against PC3 (IC_50_ 133.2 ± 5.73 μg/mL) and MDA-MB-231 lines (IC_50_ 156.9 ± 2.83 μg/mL) which could be due to the difference in chemical contents and their concentration in the two extracts.

Apoptosis plays an essential role in cellular homeostasis; nevertheless, abnormal apoptosis is a pathological process (Xiang et al. 2016). Tumour formation is the result of the loss of balance between cell proliferation and apoptosis. Induction of apoptosis is known to be a promising strategy to treat cancer (Schulze-Bergkamen and Krammer 2004). Previous research has shown that the antiproliferative effect of naturally occurring products is associated with the induction of apoptosis in cancer cells (Chidambara Murthy et al. 2011; Park et al. 2011; Zhong et al. 2011). To determine whether the antiproliferative effect of EAELN and EAELT was due to apoptosis, treated PC3 cells were stained with Annexin V and PI and analysed by flow cytometry. Analysis of PC-3 cells treated with EAELN and EAELT suggested apoptotic activity, evidenced by the accumulation of early and late apoptotic cells.

The present study showed that EAELN and EAELT induce a significant antiproliferative effect associated with apoptosis. The apoptotic effect of EAELN on the PC3 line was higher than that of EAELT (Figures 3A, B and 4A, B). The effect induced by EAELN can be attributed to its major bioactive compounds and other compounds present in the extract by a direct and also synergistic effect. These data strongly support previous research on *Linum* species as new sources for candidate anticancer drugs (Amirghofran et al. 2006; Mohammed et al. 2010; Alejandre-García et al. 2015; Akbari Asl et al. 2018).

The cell cycle plays a primary role in controlling cancer cell proliferation and cell cycle dysregulation is a fundamental feature of cancers (Vermeulen et al. 2003; Otto and Sicinski 2017). Natural products that can disrupt the cell cycle are the most commonly used anticancer drugs (Paier et al. 2018).

Therefore, the ability of EAELN and EAELT to block the cell cycle of PC3 cells was examined. Our results showed that EAELN induced a significant cell cycle arrest in the G2/M phase (Figure 4A, B), while an arrest in the G0/G1 and G2/M phases was observed in PC3 cells after exposure to various concentrations of EAELT, for 24 h (Figure 5A, B). The two extracts exhibited a different cell cycle arrest pattern.

The different observed patterns of cell cycle arrest as a pharmacological endpoint indicate the involvement of several mechanisms of action, suggesting the involvement of compounds in each extract in mediating this activity. Our results support the idea that the extracts inhibit cancer cell proliferation by inhibiting cell cycle progression, raising the possibility that the extract may be a potential therapeutic agent.

In this study, we showed, for the first time that, EAELN and EAELT exert anticancer activity through cell cycle arrest and induction of apoptosis. Interestingly, although both species belong to the same genus, the anticancer effect of EAELN was higher than that of EAELT. It was, therefore, necessary to conduct chemical analyses to reveal the molecules responsible for this activity. Therefore, LC-HRMS/MS analyses were necessary to investigate the structure-activity relationship and to compare the constituents of the two extracts. LC-HRMS/MS analyses of EAELN and EAELT are reported here for the first time, the profiling of these extracts showed the presence of compounds known for their anticancer activity. The anticancer activity induced by EAELN might be due to the major compounds detected namely, chlorogenic acid (8.19%), vicenin-2 isomer 1 (8.08%), vitexin (7.53%), trihydroxyflavone (6.70%), homoorientin (5.58%), vicenin-2 isomer 4 (5.17%), vicenin-2 isomer 2 (4.75%), vicenin-2 isomer 3 (4.67%), podophyllotoxin-β-d-glucoside (4.41%), melibiose (3.97%), cirsiliol (3.52%), tricin 5-glucoside isomer 1 (3.38%), 5,3*′*-dihydroxyflavone (2.80%), luteolin (2.61%), and orientin isomer 1 (2.54%) (Table 2 and Figure 7A).

Similarly, the anticancer activity induced by EAELT might be due to the presence of major compounds namely, 4-caffeoylquinic acid (10.51%), homoorientin (9.21%), cirsiliol (8.24%), chlorogenic acid (7.44%), 3-*O*-coumaroylquinic acid isomer 2 (4.63%), tetrahydroxyflavone (4.36%), 2ʺ-*O*-β-d-xylopyranosylorientin (4.05%), hyperoside (3.87%), 3-*O*-feruloylquinic acid isomer 2 (3.85%), 4,5-dicaffeoylquinic acid isomer 2 (3.55%), vicenin-2 isomer 1 (3.50%), quercitrin (3.35%), 4,5-dicaffeoylquinic acid isomer 1 (3.05%), methyl chlorogenate isomer 1 (2.97%), and vitexin (2.93%) (Table 2 and Figure 7B).

The anticancer effect of EAELN was higher than that of EAELT, this could be explained by its richness in chicoric acid (8.19%), vicenin-2 isomer 1 (8.08%), vitexin (7.53%), and podophyllotoxin-β-d-glucoside (4.41%) compared to EAELT, which presented lower levels of these compounds known for their anticancer activities. Indeed, it has been reported that chicoric acid has a strong growth inhibitory effect against HCT-116 colon cancer cells and effectively induces apoptosis, characterised by DNA fragmentation, caspase-9 activation, PARP cleavage, and β-catenin downregulation (Tsai et al. 2012). Its anticancer activity on the MCF-7 cell line has also been noted (Huntimer et al. 2006). Vicenin-2 has also been reported to have numerous pharmacological properties, including antioxidant, anti-inflammatory and anticancer effects (Ku and Bae 2016; Yang et al. 2018). Studies by Nagaprashantha et al. (2011) have also shown that vicenin-2 induces antiangiogenic, pro-apoptotic and antiproliferative inhibition of prostate cancer cells. Vicenin-2 also inhibits HT-29 colon cancer cell proliferation by inhibiting the WNT/β-catenin signalling pathway, inducing apoptosis, and leading to the arrest of HT-29 cells in the G2/M phase. Furthermore, treatment with vicenin-2 increases the expression of apoptosis-associated proteins Bax, cytochrome c, and caspase-3, and decreases that of Bcl-2 (Yang et al. 2018). Vitexin is a naturally occurring flavonoid compound that exhibits antioxidant (An et al. 2012), anticancer and neuroprotective properties (Zhu et al. 2016). Recently, vitexin has attracted the attention of many researchers for its potential antitumor properties. This compound has shown an antitumor effect against various cancers, including breast, prostate, and ovarian cancers, by inhibiting proliferation and promoting apoptosis of cancer cells (Zhou et al. 2009; He et al. 2016; Ganesan and Xu 2017). Zhou et al. (2009) reported that vitexin inhibited the proliferation of prostate, breast and ovary cancer cells and induced apoptosis by activating caspases and decreasing the Bcl-2/Bax ratio. Vitexin has been shown to induce G2/M phase cell cycle arrest and apoptosis by affecting the Akt/mTOR signalling pathway in human glioblastoma cells and non-small cell lung carcinoma (Zhang et al. 2018; Liu et al. 2019). Moreover, podophyllotoxin glycosides and their derivatives are considered to lead compounds for anticancer drug development. The podophyllotoxin-β-d-glucoside compound exhibits more potent cytotoxic activity than the control drug (etoposide) in various cancer cell lines (PC-3, HeLa, HCT-116, HEK-293, and MCF-7) (Zilla et al. 2014). It has also been noted for its cytotoxic activity in other human cancer cell lines, HL-60, SMMC-7721, A-549, and SW480 (Zi et al. 2019). Podophyllotoxin and its derivatives exhibit anticancer activity, mainly due to its ability to inhibit tubulin polymerisation into microtubules (Kamal et al. 2014). It is obvious that the observed anticancer activity of the extract could be due to one or a mixture of the compounds highlighted above. Moreover, *in silico* studies regarding the interaction of the identified compounds with potential biological targets involved in cell proliferation could provide valuable information.

## Conclusions

Our results demonstrated for the first time that ethyl acetate extracts of two *Linum* species, *L. numidicum* (EAELN) and *L. trigynum* (EAELT), inhibited the proliferation of PC3 and MDA-MB-231 cells in a dose-dependent manner. EAELN had the highest antiproliferative activity against both lines tested. EAELN and EAELT induced apoptosis of PC-3 cells; the apoptotic effect of EAELN was higher than that of EAELT extract. EAELN induced a significant cell cycle arrest in the G2/M phase, while an arrest in the G0/G1 and G2/M phases was observed after treatment with EAELT. In this study, we showed, for the first time, that EAELN and EAELT exert anticancer activity by inducing apoptosis and blocking the cell cycle. This could be due to the presence of phenolic compounds such as flavonoids, lignans and phenolic acids which were detected by LC-HRMS/MS. The anticancer effect of EAELN was higher than that of EAELT. The effect induced by EAELN could be attributed to its major bioactive compounds such as chicoric acid, vicenin-2, vitexin, and podophyllotoxin-β-d-glucoside and other compounds present in the extract by a direct and/or synergistic effect. EAELN can be consered as a source of phytochemicals to treat cancer. Owing that most of the detected compounds can be obtained from commercial sources, it is intended to test those of them that are known for their anticancer activity.
